# Innovating teaching and instruction in turbulent times: The dynamics of principals’ exploration and exploitation activities

**DOI:** 10.1007/s10833-022-09458-2

**Published:** 2022-05-24

**Authors:** Marcus Pietsch, Pierre Tulowitzki, Colin Cramer

**Affiliations:** 1 Leuphana University of Lueneburg, Universitätsallee 1, 21335 Lüneburg, Germany; 2 University of Applied Sciences and Arts Northwestern Switzerland, Bahnhofstrasse 6, 5210 Windisch, Switzerland; 3 Eberhard Karls University of Tübingen, Wilhelmstrasse 31, 72070 Tübingen, Germany

**Keywords:** Ambidexterity, COVID-19, Exploitation, Exploration, Innovation, Knowledge, Principals

## Abstract

In turbulent environments, schools have to adapt to constantly changing conditions. According to ambidexterity theory, whether they are successful in this primarily depends on their leaders and how they manage the tension between the use of current knowledge (exploitation) and the search for new knowledge (exploration). Through unique top-down and bottom-up pathways, they thus influence the innovation outcome of a school. However, it is so far unclear whether these assumptions are correct. Using data from a panel of principals who are representative of Germany and were surveyed before and during the COVID-19 pandemic, we therefore investigate if and how school leaders adapted to the turbulent environment caused by the pandemic and evaluate the extent to which this had an impact on their schools’ innovations in teaching and instruction. The results demonstrate that principals’ exploration activities increased markedly during the pandemic, while their exploitation activities decreased noticeably. Further, a focus on the use and refinement of existing knowledge in comparatively predictable (pre-COVID-19) environments harmed principals’ readiness to explore new knowledge in increasingly uncertain environments. Nevertheless, exploitation had positive consequences for the innovativeness of schools, and exploration goes along with more radical innovations in teaching and instruction. Our research suggests that schools that innovatively addressed the COVID-19 pandemic had school leaders who were able to quickly shift between the two modes of exploitation and exploration. A capacity to transition seamlessly between these modes of thinking and working thus appears to be vital for the longevity of schools.

## Introduction

The COVID-19 pandemic has posed a massive challenge for education systems across the globe. During the first peak period in April 2020 alone, schools were closed nationwide in 190 countries, with more than 90% of the world’s students affected by these closures as a consequence (UNESCO, 2020). Never before have schools had to deal with so much uncertainty and faced such unexpected and unique challenges on a global scale. Accordingly, Audrey Azoulay, Director-General of UNESCO, called the COVID-19 crisis “the most unprecedented disruption in the history of education” (UNESCO, 2020, p. iii).

It would be hard to describe more aptly the situation that schools faced during this pandemic, particularly as the abrupt suspension of face-to-face teaching and learning in the classroom could have led to schools, or at least their core processes of teaching and learning, ceasing to operate entirely (Viner et al., 2020). This was particularly so because the move to remote learning environments was unfamiliar to many schools, was often not an exigency for which schools had prepared, and exposed wide gaps in access to technology (Kuhfeld et al., 2020). In this respect, the pandemic and the resulting school closures constituted extreme turbulence for schools (Beabout, 2012), an environment that had the potential to cause “structural damage to the institution’s normal operation” (Gross, 2014, p. 260).

All professionals in school had to deal with these extremely challenging circumstances, and it was particularly incumbent on principals to navigate their schools through these turbulent times and ensure their continued functioning (Harris & Jones, 2020). As leaders, they had to balance reducing uncertainty for schooling on the one hand (Weiner et al., 2021) and immediately creating and launching new ways of teaching and learning on the other (Harris & Jones, 2020). In order to achieve both aims, principals had to ensure that educational processes continued to operate while applying a “messy, trial-and-error, butterflies-in-the-stomach leadership” (Harris, 2020, p. 324; see also Munby, 2019, p. 2).

In the management and organizational literature, some scholars use the term exploitation–exploration paradox for this kind of tension (Andriopoulos & Lewis, 2008), with exploitation referring to incremental improvements in and refinement of school activities and their leaders and exploration relating to experimentation and radical innovation (Bingham & Burch, 2019; Pietsch et al., 2020). Ambidexterity theory assumes that organizations and their leaders need to shift between these two complementary, mutually affecting knowledge strategies on an ongoing basis to secure the functioning and survival of the organization—and thus to act ambidextrously—particularly in dynamic and turbulent environments (Benoliel & Schechter, 2017; Bingham & Burch, 2019; Da’as, 2021, 2022; Pietsch et al., 2020).

To date, however, no study has examined the relationship between exploration and exploitation and the implementation of innovations in learning and teaching, either before or during the COVID-19 pandemic. As a result, although many studies in other research fields point to a relationship between the exploitation and exploration activities of leaders and innovation (Gieske et al., 2020; Guisado-González et al., 2017; Jansen et al., 2005; Rosing & Zacher, 2017), there is currently no evidence to draw corresponding conclusions in the school context. Moreover, even in general ambidexterity research, the relationship between individual ambidexterity and the innovation performance of organizations has hardly been investigated so far (Pertusa-Ortega et al., 2021). Thus, in this paper, we explore the question of whether and to what extent the exploitation and the exploration activities of principals before and during the pandemic fostered innovation in teaching and instruction during pandemic-related school closures.

For this purpose, we analyze data from the Leadership in German Schools (LineS) study, a principal panel (*N* = 493) that is representative of Germany. The study aims to research the careers of school leaders in Germany for the first time using a representative random sample on a longitudinal basis. In order to investigate the disruption that accompanied the COVID-19 pandemic and the role of school leaders in this context, a special survey was conducted at short notice at the beginning of the pandemic as part of the panel. Hence, the data used were collected at two measurement points within one school year; in autumn 2019, about 6 months before all schools were closed in Germany due to COVID-19, and in spring 2020, during that nationwide school closure. Based upon this data, we seek to understand (a) if principals’ exploitation and exploration activities were affected by the turbulent environment caused by COVID-19 and (b) if the dynamic duality between exploitation and exploration is associated with a schools’ innovativeness on the level of teaching and instruction. One aim of our article therefore is to interrogate the dynamics of principals’ exploitative and explorative activities over time and to investigate whether and how principals adapted their activities to the changed environment. A second aim is to investigate possible longitudinal effects of principals’ exploitation and exploration on innovation of teaching and instruction in the extremely turbulent environment caused by COVID-19.

## Background and conceptual grounding

### Conceptualizing exploitation, exploration, and ambidexterity

Exploitation and exploration are two different types of organizational and individual adaptation to the environment (Lavie et al., 2010; March, 1991). Exploitation refers to activities that capitalize on knowledge and competencies already available to the organization or individual; in other words, it works within and refines the familiar frame of reference (March, 1991). Exploration encompasses activities like experimenting and innovating in pursuit of new knowledge. Explorative activities are therefore prone to uncertainty and failure but can also lead to disruptive innovations that can change the status quo, even in organizations with comparatively limited resources (Christensen et al., 2015). On the individual level, explorative activities are characterized by thinking outside the current frame of reference and beyond the currently accepted ways of doing things (Good & Michel, 2013).

Thereby, the exploration–exploitation distinction exhibits common features with many other concepts in the field of organizational learning, but points to the tension between different forms of learning and the corresponding dynamics (Papachroni et al., 2015). For example, some authors (e.g., Brix, 2019; Lam, 2019; Papachroni et al., 2015) equate exploitation with single-loop or adaptive learning and exploration with double-loop or generative learning in the sense of Argyris and Schön (1978) and Senge (1990). However, while classical concepts assume that organizations and their leaders usually have to make either/or decisions about which learning strategy to use March’s (1991) concept of exploration and exploitation views the different learning modes as dualities that influence each other but need to be actively managed on an ongoing basis (Gibson & Birkinshaw, 2004; Lam, 2019; Lewis, 2000). Therefore, the focus here is on “managerial and organizational flexibility” (Lavine, 2014, p. 191) and on the “continuous developing and changing of the exploration–exploitation configuration” (Krause-Söhner, 2021, p. 32) in ever-evolving contexts.

Accordingly, exploitation and exploration are fundamentally different logics that create tension because they require different modes of operation and different resource allocations and compete for scarce resources (March, 1991). On the individual level, leaders must be able to manage tensions between these two knowledge strategies and (repeatedly) reallocate individual resources accordingly (O’Reilly & Tushman, 2011). Hence, dealing with these contradictory demands may lead to cognitive strain (Keller & Weibler, 2015) and stress in them (Hunter et al., 2017). Both modes can be considered dynamic capabilities (O’Reilly & Tushman, 2008) and complementary, mutually affecting forces (Raisch & Zimmermann, 2017) that need to be carefully balanced to achieve organizational success over time (Raisch et al., 2009). The corresponding theoretical concept is called ambidexterity, which can be understood as the ability of an organization or an individual within an organization to pursue exploitation and exploration simultaneously (Mom et al., 2009; O’Reilly & Tushman, 2004).

### The dynamics of exploitation and exploration

Ambidexterity has been reported to be vital for the longevity of an organization (Gibson & Birkinshaw, 2004), particularly in competitive, more dynamic, and unpredictable contexts where the likelihood of a disruptive change is higher (Tushman & O’Reilly, 1996). The original understanding of ambidexterity was being able to manage these concurrent processes effectively by balancing the competing demands of exploration and exploitation (March, 1991). In March’s view (1991, p. 105), “the basic problem confronting an organization is to engage in sufficient exploitation to ensure its current viability and, at the same time, devote enough energy to exploration to ensure its future viability” and thus is about continuously balancing two ends of a continuum. However, Gupta et al. (2006) proposed a more sequential perspective, viewing ambidexterity as being able to shift rapidly between exploration and exploitation. Viewed in this vein, principal ambidexterity can be understood as dynamic dualism between these two knowledge strategies, “whereby stability may enable change, and change may enable stability” (Papachroni & Heracleous, 2020, p. 17) and consequently one activity can strengthen the other if both are connected through learning (Cao et al., 2009). For instance, members of an organization might come up with new ideas even when working within familiar knowledge frames (Cao et al., 2009). By the same token, exploration might be able to strengthen exploitation by generating additional complementary resources (Cao et al., 2009). For example, an innovation stemming from exploration might not only lead to new ways of doing things in an established area (exploitation), but also in another, unexpected and possibly unrelated one. In organizational research, the dominant view is that exploitation is more favored by management due to its promise of short-term gains (Levinthal & March, 1993). This risks an organization becoming “stuck” on a path that revolves mostly around exploitation, which is known as exploitation bias:

The competition for resources is asymmetric, with exploitation innovations harming exploration innovations but not the other way around. The result is that performing exploitation does not merely improve an organization’s exploitation routines and increases the likelihood that exploitation will be performed again; it also reduces the resources available for exploration (Greve, 2007, p. 953).

While an exploitation bias is more common, any focus that becomes too dominant and rigid can put an organization at risk (Keller & Weibler, 2015). For example, leaders in an organization that seems to perform well might focus more on maintaining and optimizing the current state of affairs, which becomes the dominant area of attention, with hardly any attention paid to innovation and generating new knowledge. This is referred to as the competency trap (Levinthal & March, 1993). Such an organization may not be able to adapt to a disruptive change. The counterpart to the competency trap is the failure trap, in which leaders focus too much on exploration, constantly chase new ideas, thus creating (in the worst case) a cycle of failed innovations without reaping any benefit (Levinthal & March, 1993). The core issue with both these extremes is the inability to change modes dynamically, which is referred to as path dependency. A major challenge therefore is not only achieving but also maintaining ambidexterity, staying dynamic and mindful of both exploration and exploitation in ever-evolving contexts. Ambidexterity as a dynamic capability allows one “to overcome inertia and path dependencies [and] is at the core of dynamic capabilities” (O’Reilly & Tushman, 2008, p. 187). Overcoming both exploitation bias and path dependency is crucial for being able to adapt in the face of the unexpected (Andriopoulos & Lewis, 2008).

### Innovating education in turbulent times

Already in his foundational work, March (1991) linked the tension between exploration and exploitation to innovation, noting that exploitation is primarily associated with efficiency and refinement, while exploration is primarily associated with innovation and experimentation. Consequently, both modes are associated with change, with exploitation leading to gradual, cumulative change and exploration leading to discontinuous, radical change (Maclean et al., 2021). In this understanding, all innovation is change, but not all change involves innovation (Osborne & Brown, 2005). Accordingly, an organization’s innovation outcome is the result of exploration and exploitation and can be measured by the extent to which an innovation differs from existing alternatives in its degree of newness and novelty (Damanpour & Aravind, 2012), more specifically its innovation radicalness (Johannessen et al., 2001). This corresponds to the understanding of the OECD, which defines innovation in organizations in its guidelines for collecting, reporting, and using data on innovation as follows:

An innovation is a new or improved product or process (or combination thereof) that differs significantly from the unit’s previous products or processes and that has been made available to potential users (product) or brought into use by the unit (process). (OECD & Eurostat, 2018, p. 60).

In ambidexterity research, leaders are seen as the key drivers in addressing the tension between exploration and exploitation and its relationship to organizations’ innovation (Mom et al., 2019; Rosing & Zacher, 2017; Smith & Tushman, 2005; Zimmermann et al., 2018). Ambidexterity is considered here as dynamic managerial capability (Papachroni & Heracleous, 2020) that enables leaders to manage complex organizations (Smith & Lewis, 2012; Smith et al., 2010) and promote ambidexterity at both team and organizational levels (Jansen et al., 2016) through interactions across organizational levels (Mom et al., 2019) ultimately leading to positive effects in organizational performance (Eisenhardt et al., 2010), such as an organization’s innovation outcome (de Visser & Faems, 2015).

Research in business organizations indicates that “turbulent environments favor organizations that can promptly take advantage of emerging opportunities and abandon expiring certainties” (Lavie et al., 2010, p. 119), that such organizations allocate more resources toward exploration activities during turbulent times (Lant & Mezias, 1992), and that the rate and radicalness of innovations can significantly increase as a consequence (Germain, 1996). In particular, leaders have been shown to drive more radical changes in increasingly uncertain environments (Koberg et al., 2003). Consequently, the ambidexterity of leaders is particularly useful for organizational performance and innovativeness in unpredictable and highly dynamic contexts (Good & Michel, 2013).

Compared to business organizations, however, schools have repeatedly been characterized as rather resistant to fundamental change; Tyack and Tobin (1994) identified a “grammar” of schooling in long-standing structures (e.g., subject-based instruction, age-based classes, fixed lesson schedules) that influences many aspects of schooling and effectively “absorbs” many innovative efforts. The reasons for this are varied; structural characteristics of schools are themselves are limiting, schools serve multiple constituents making changes hard to plan and predict and they are responsible for passing down civic and cultural knowledge and thus have a certain obligations to preserve the past (Tye, 2000). The continuity of the school system therefore can also be viewed as a way to protect the current strengths of the system. Some scholars have started to reject the notion of ‘continuity vs. innovation’ and argued that schools and school systems can be considered hybrids, maintaining “stability by adopting incremental changes” (Cuban, 2020, p. 669).

When it comes to challenging the grammar of schooling and driving innovation on the classroom level, school leaders play a key role (Hubbard & Datnow, 2020). As they can influence key characteristics of the school—the vision, structures and processes, and working conditions and staff capacity (Hallinger, 2011; Leithwood et al., 2020)—they can act as drivers of educational change (Harris et al., 2013). It has been shown that principals’ ambidexterity does affect teaching and learning by promoting a climate that supports teacher creativity and consequentially teachers’ classroom practices (Da’as, 2021). Furthermore, the pandemic highlighted the general relevance of school leaders in creating conditions of psychological safety and for innovation in times of crisis (McLeod & Dulsky, 2021; Weiner et al., 2021). In a similar vein, Beabout argues that in times of crisis, “change is also dependent on that system having enough stability for members to safely experiment with new ways of doing things while remaining grounded in the safety of a recognizable system” (Beabout, 2010, p. 419).

## The present study

### Hypotheses

Based on the above literature review and the lack of empirical studies in the educational field in this regard, we test the following hypotheses.

#### Change of resource allocation in turbulent times

##### H1a

Exploitative activities of principals decrease over the course of the COVID-19 pandemic.

##### H1b

Explorative activities of principals increase over the course of the COVID-19 pandemic.

#### Path dependency

##### H2a

Exploitative activities of principals prior to the COVID-19 pandemic are positively associated with exploitative activities of principals during the COVID-19 pandemic.

##### H2b

Explorative activities of principals prior to the COVID-19 pandemic are positively associated with explorative activities of principals during the COVID-19 pandemic.

#### Competency and failure trap

##### H3a

Exploitative and explorative activities of principals are associated over time, meaning that exploitative activities prior to the COVID-19 pandemic improve or reduce exploration activities during the COVID-19 pandemic.

##### H3b

Explorative and exploitative activities of principals are associated over time, meaning that explorative activities prior to COVID-19 pandemic improve or reduce exploitation activities during the COVID-19 pandemic.

#### Association of exploration and innovativeness

##### H4

Explorative activities of principals prior to and/or during the COVID-19 pandemic are positively associated with a school’s innovativeness, specifically the creation of new teaching and instruction processes, during the COVID-19 pandemic.

#### Association of exploration and innovation radicalness

##### H5

Explorative activities of principals prior to and/or during the COVID-19 pandemic are positively associated with their schools’ process innovation radicalness, specifically the degree of novelty in teaching and instruction processes, during the COVID-19 pandemic.

### Study context

How a school system deals with the challenges posed by the COVID-19 pandemic depends to a large extent on structures that have evolved in national or state school systems. In this respect, the German school system can be described as rather conservative with regard to innovation in comparison with its Scandinavian neighbors (Groß Ophoff & Cramer, 2022). This applies not only to the use of research for innovation in schools and teaching but also to the slow pace of equipping schools with up-to-date digital infrastructure. About €5 billion in federal funds for digitizing schools were made available already before the pandemic began. These funds were initially only drawn down to a small extent by schools, which had to submit an application justifying their need for and intended use of the funds (Drahmann et al., 2020). With the advent of the pandemic, many schools appeared unable to cope with the increased demands on their digital infrastructure and subsequently failed to be in direct contact with students digitally or to teach synchronously using video tools. But even at the beginning of the pandemic, the funds were drawn down only hesitantly. Obviously, the potential of digital media for distance learning was initially underestimated or schools at first relied on digital equipment in the private households of families. These recent experiences exemplify the fact that school leaders in Germany have traditionally tended to focus on their administrative tasks, i.e., ensuring schooling by using the already available means and resources (exploitation), which are associated with a high workload, because they are finally not suitable for achieving this goal. Radical innovations (exploration) have often been missed, such as a consistent expansion of the digital infrastructure of schools, because space for innovation is rare, which in the pandemic finally was proving to be a problem (Pietsch et al., 2020) and could even further increase exploitative activities to compensate for earlier omissions (exploitation bias).

In the Federal Republic of Germany, education is the responsibility of 16 federal states, so the structures and organization of school systems and teacher education differ (Terhart, 2019). After compulsory education at primary schools for students from ages 6 to 10 in all states, students of different abilities are tracked into one of several types of school, which usually differ in both duration and curriculum. While German states traditionally have three different secondary school types in lower secondary education and an additional upper secondary school, educational reforms have led most states to introduce at least one comprehensive secondary school. In this still highly differentiated school system, the COVID-19 pandemic is only a catalyst that makes more virulent the problems that arose from the school systems and teacher education of past decades.

The German school system has consistently been oriented to face-to-face teaching. Except for homework and exam preparation, there is hardly any experience with distance learning and even less with using digital media. For example, the International Computer and Information Literacy Study (ICILS) found that students in Germany spend very little time learning with digital media and that relevant equipment and competences are lacking by international comparison (Eickelmann et al., 2019; Fraillon et al., 2020). Depending on their parents’ social background, the students do not always have the necessary digital infrastructure with fast internet and suitable digital devices, and their schools do not provide them with all the appropriate devices as a matter of course. Even teachers are generally not provided computers and are left on their own when it comes to digital media. In teacher education, too, systematic engagement with content such as digital teaching and learning has only recently found its way into the compulsory curriculum in university courses. Although all students use digital devices in their studies, didactic issues surrounding the use of digital media and tools in schools and classrooms continue to play only a marginal role in teacher education.

Deficits in the digital infrastructure of schools pose a particular challenge under pandemic conditions. In Germany, as the number of infections increased, all schools were completely closed for attendance in mid-March 2020, with only a few children offered emergency care. As a result, distance learning at home had to be implemented nationwide and monitored. Because education with school attendance is compulsory for all children, home schooling (also known as home education) is a novelty in Germany; indeed, it is legally prohibited and a matter of controversy (Spiegler, 2009). It was not until mid-May 2020 that the schools gradually reopened to face-to-face teaching, alternating small groups of students. The inadequate digital infrastructure, especially for socially disadvantaged children, was an enormous challenge, and not all schools had the necessary number of digital devices to equip all children. As a result, a number of students in home schooling were unable to follow synchronous digital learning via video conferencing, so assignments and work materials also had to be sent by regular mail or picked up and dropped off at schools.

In view of these failures, the extent to which schools in Germany have so far been able to build up a digital infrastructure and use digital media in their everyday work depends on their individual commitment. School leadership is of particular importance in this context, as it has a significant influence on the extent and implementation of digitization at a given school. There are likely to be significant differences between the degree to which individual school leaders advanced digitization and thus the conditions for distance learning before the pandemic and how quickly they were able to respond to the demands posed by COVID-19. In particular, school leaders with the ability to anticipate the necessary innovations and respond quickly to requirements are likely to have advantages in dealing with the pandemic-related challenges. However, the qualification paths of school leaders in Germany are very diverse and unsystematic (Tulowitzki et al., 2019), and preparation for digital challenges has thus far played only a minor role (Cramer et al., 2019).

### Sample and procedure

Our study relies on a randomized and nationally representative panel of German principals who responded to online questionnaires during two waves within a single school year (thus far). The underlying population for the data consists of all principals in Germany working at schools of all types. The data from the first wave, the primary sample, were gathered between August and November 2019 by *forsa* GmbH, a leading German survey firm, using a piloted and standardized online questionnaire and comprised *N* = 405 principals. The data from the second wave were gathered in the same school year, between mid-April and mid-May 2020, during the period when all schools in Germany were closed due to the pandemic. All panel members were recruited using a multi-stage random process within *forsa’s* daily omnibus survey, in which a sample of 1000 people over the age of 14, representative of Germany, is randomly interviewed by telephone on various topics every working day. Thus, in a first step, within the framework of this survey, a sub-sample of school principals was determined by means of screening and then given an individualized link to the online survey. In a second step, this random sample of school principals, also representative of Germany, answered the questionnaire we developed and made available online by *forsa*.

To handle potential panel attrition, a refreshment sample (Deng et al., 2013; Hirano et al., 2001; Taylor et al., 2020) of *N* = 88 (> 20% of the primary sample) principals was sampled by *forsa* in wave two, applying the same criteria and the same procedure as in wave one. This was also due to the fact that the second survey was not originally planned at this time and therefore took place at short notice and unexpectedly for the participants. *N* = 218 of the principals completed the questionnaires during both waves, *N* = 187 principals provided information during only the first wave, and *N* = 88 principals, the refreshment sample, answered questions only during the second wave. To minimize common method biases, we followed the procedural suggestions of Podsakoff et al. (2012) during both waves. Thus, for example, we varied item wordings and scale properties across different scales and scrambled and personalized both individual items and item blocks throughout the surveys.

In our total sample, 55.8% of the principals were female, 43.8% were male and 0.2% did not provide gender information. The mean age was 53.54 years (SD: 7.72). On average, principals worked as teachers for 15.11 years (SD: 7.25) before becoming principals and had been in a leadership position for 9.94 years (SD: 7.31) at the time of the survey. In addition, 91.5% of the principals worked in public schools and 8.5% worked in private schools. Respondents indicated that they have changed jobs 2.86 times (SD: 1.94) so far and that they work a total average of 48.72 h per week (SD: 9.05), of which they teach 11.27 h per week (SD: 5.77) in addition to their leadership activities.

### Measures

We measured *exploitation and exploration* by applying items and scales developed by Mom et al. (2009). Thus, regarding the first wave, the exploration scale determines the extent to which a principal engaged in exploration activities during the previous year, while the exploitation scale determines the extent to which the principal engaged in exploitation activities during the previous year (Base question: “To what extent did you, during the last 12 months, engage in work-related activities that can be characterized as follows?”). In the context of the longitudinal study, four items of the original six-item scale (see ‘Appendix 2’) were applied during both waves as anchor items, two per dimension. Thus, during the first wave the principals answered two items measuring exploitation behaviors or activities characterized by focusing attention on refining existing knowledge and skills and implementing existing plans (e.g., “Activities which you can properly conduct by using your present knowledge”). Here, our indicator of internal consistency, McDonald’s Omega (*ω*, McDonald, 1999), was *ω* = .69. They then answered two items indicating exploration (*ω* = .76), behaviors or activities in the school context associated with fewer certainties and a higher risk for failure (e.g., “Activities requiring you to learn new skills or knowledge”).

Regarding the second wave, the exploration scale determines the extent to which a principal engaged in exploration activities since his or her school was closed by the COVID-19 pandemic, while the exploitation scale determines the extent to which a principal engaged in exploitation activities during that same period (Base question: “To what extent did you, since your school closed due to COVID-19, engage in work-related activities that can be characterized as follows?”). The internal consistency at the second measurement point was *ω* = .75 for exploitation and *ω* = .75 for exploration. All items were measured on a four-point Likert scale ranging from “a very small extent” to “a very large extent” of engagement in either explorative or exploitative activities.

*Innovation* was measured by adapting items and scales from the European Community Innovation Survey (CIS, Behrens et al., 2017), which is based on the aforementioned definition from the OECD’s & Eurostat (2018) Oslo guidelines for collecting, reporting, and using data on innovation. As Arundel et al. (2019) state, the OECD definition encompasses a broad range of innovations, from minor incremental improvements to disruptive or transformative innovations that completely alter or replace processes or services. The guidelines also distinguish between product, process, marketing, and organizational innovations.

Accordingly, with regard to teaching and instruction, which we understand to be the core processes of schooling, we provided the following description to the principals in terms of process innovations: “Process innovations are new or noticeably changed processes with regard to the pedagogical work of the school (e.g., instruction and/or teaching).” Next, the principals were asked if their school had introduced such new or significantly improved processes since school closure was implemented (item: “Did your school introduce new or significantly improved processes since the school closed?”), with answers binary coded as 0 = no, 1 = yes. Subsequently, principals were asked in an open-ended question to name and describe these innovations (item: “What were the main innovations in this area during the school closure? Please give a maximum of three examples”). Finally, they had to specify the innovation radicalness of these innovations (item: “Are these changes incremental (improving and/or supplementing and/or adapting what already exists) or radical (introducing something completely new) for your school?”) on a ten-point Likert scale.

Because several contextual factors could influence the ambidexterity of principals and the innovation capacity of their schools, we use the also collected as part of the survey information below to control for possible confounding effects:

*School type* applies the International Standard Classification of Education (ISCED; UNESCO Institute for Statistics, 2012), which distinguishes education systems according to uniform criteria: ISCED 1 refers to “primary education” and covers the 1st to 4th school years in Germany, ISCED 2 refers to “lower secondary education” and covers the 5th to the 10th years, and ISCED 3 refers to “higher secondary education” and covers the 11th to 13th years. Thus, in our study we differentiate between primary schools, secondary schools, special needs schools, and other schools (mainly schools with both primary and secondary branches). We constructed four dummy-coded variables (coded 0 and 1) and defined primary schools as the reference group. Within our sample, 51.3% are leaders of primary, 38.9% are leaders of secondary, 6.7% are leaders of special needs, and 3.0% are leaders of other schools.

*School size* is measured by the total number of students enrolled in a school. This variable was added to our analyses partly because school size may affect interpersonal distance and organizational structures (Bush, 2010), which may be relevant to a principal’s choice of management and leadership practices. In addition, the size of a school’s student body might be associated with its innovation capacity (Preston et al., 2012). Within our sample, school sizes ranged from 25 to 2000 students enrolled, with a mean of 360.83 (SD = 299.64).

*School location* or *rural–urban split* refers to the urban or rural character of the area in which a school is situated. We control for this variable because urban and rural schools may differ with regard to infrastructure, especially internet connections, and other factors that might affect innovation (Bouck, 2004). To survey the urban–rural profile, we applied an item from PISA 2012 (OECD, 2013): “Which of the following definitions best describes the community in which your school is located?” Within our sample, 87 schools (17.6%) were in a village, hamlet or rural area (fewer than 3000 people), 160 (32.5%) in a small town (3000 to about 15,000 people), 158 (32.0%) in a town (15,000 to about 100,000 people), 66 (13.4%) in a city (100,000 to about 1,000,000 people), and 21 (4.3%) in a large metropolitan city (over 1,000,000 people).

### Analytical strategy

As we are interested in the dynamic effects of principal ambidexterity on innovation in teaching and instruction during the COVID-19 pandemic and potential path dependencies of principal exploration and exploration, we scrutinized latent cross-lagged panel models (CLPMs, Zyphur et al., 2020a) in MPlus 8.3 (Muthén & Muthén, 2017). These models provide two types of coefficients: First, autoregressive paths that provide information about inter-individual differences in one variable over time, in this case the stability of exploitation and exploration between the two measurement points, and second, cross-lagged paths that provide information about the relation of two (or more) different variables over time and that make it possible to examine whether a predictor variable accounts for a change in another longitudinal observed variable. As these associations enact a temporal order, the panel coefficients can be interpreted as causal influences (Little, 2013).

When CLPMs are fitted, invariance for modeled factors, here exploration and exploitation, over time is assumed (Xu et al., 2020); thus, it is important to ensure that the cross-lagged relationships investigated are not biased by the instability of the factor structure of latent variables across time points (Widaman et al., 2010). Hence, we successively tested for factorial measurement invariance (Meredith, 1993), as longitudinal measurement invariance can be evaluated at four levels, ranging from weak to strong (Widaman et al., 2010): configural, metric, scalar, and strict invariance. Configural invariance (i.e., factor structures are the same over time) is the weakest, while strict invariance (i.e., factor loadings, thresholds, and residuals are the same across time points) is the most restrictive. *Configural invariance* means that constructs are indicated by the same items over time. *Metric invariance* indicates that factors over time have the same meaning, that their units and intervals are comparable, as factor loadings are equal across time points. *Scalar invariance* occurs when, in addition, item intercepts are equal and thus all items indicate the same differences in latent means over time. *Strict invariance*, finally, indicates that residual variances are the same over time in addition to the equality of factor loadings and item intercepts.

To assess the fit of the models, the comparative fit index (CFI), root mean square error of approximation (RMSEA), and standardized root mean square residual (SRMR) as provided by MPLUS are all reported. Generally, acceptable fit is indicated by a CFI over .900, an RMSEA below .080, and an SRMR less than .080 (Hu & Bentler, 1999; Marsh et al., 2004). Regarding the evaluation of invariance, we investigated changes in the CFI, RMSEA, and SRMR at each stage of testing. Here, a difference greater than .010 in the CFI (ΔCFI ≥ .010), a difference greater than .015 in the RMSEA (ΔRMSEA ≥ .015), and a difference greater than .030 in the SRMR (ΔSRMR ≥ .030) values between less constrained and more constrained models suggest a lack of invariance (Chen, 2007; Cheung & Rensvold, 2002). Additionally, we assumed invariance existed between models if changes in the CFI (ΔCFI) were less than or equal to .002 (ΔCFI ≤ .002), as Meade et al. (2008) have shown that ΔCFI is not sensitive to sample size and is adequate for sample sizes of 400 or more.

Unit non-response or panel attrition (i.e., principals that participated in wave one but not wave two of our study) between waves in our data was 46.2%; item non-response for our measures during wave one was 0.70% and 3.60% during wave two. To handle unit non-response and cross-sectional missing data (item non-response), we followed Akande et al. (2021), Deng et al. (2013), and Hirano et al. (2001) and thus combined refreshment with a multiple imputation approach (i.e., P+R approach, see Deng et al., 2013). Consequently, at each stage of analysis we generated a completed data set that included all *N* = 493 cases from the panel and refreshment sample, imputed the data 100 times, and used these data for estimating our CLPM and all other reported coefficients and statistics (see ‘Appendix 3’ for an exemplary Mplus input).

## Results

### Descriptive statistics, correlations, and univariate analyses

As shown in Table 1, exploitation and exploration at both measurement points are correlated with each other. It is notable that both modes of principal ambidexterity are negatively correlated. Thus, a trade-off is observable and is clearly more pronounced at the second measurement point, during COVID-19-related school closures (*r* = − .703, *p* < .001), than during the first measurement point (*r* = − .454, *p* < .001), about 6 months before the COVID-19 pandemic led to school closures across Germany. Frequent exploitation is thus at the expense of exploration, as school leaders have to make an either/or decision and allocate their available resources accordingly (Gibson & Birkinshaw, 2004). Moreover, all measures are significantly related with one another over time, indicating a potential path dependency for both exploration (*r* = .387, *p* = .001) and exploitation (*r* = .432, *p* < .001), as well as a persistent trade-off between exploration and exploitation over time (*r* = − .249, *p* = .019 and *r* = − .414, *p* < .001). Further, principals spent more time on explorative activities (*m* = 2.94) than on exploitative activities (*m* = 2.64) during the school closure. Thus, the proportion of the first measurement point were virtually reversed compared to the first measurement point, meaning that about 6 months before COVID-19, principals spent far more time executing exploitative activities (*m* = 3.22) than explorative activities (*m* = 2.55). Both changes are statically significant (*p* < .001), so H1a and H1b are accepted, as we found an increase in explorative and a decrease in exploitative activities of principals over time.

**Table 1:** Means, standard deviations and latent correlations of exploration, exploitation and innovation

		Mean	SD	Correlations									
				1		2		3		4		5	6
				*r*	*p*	*r*	*p*	*r*	*p*	*r*	*p*	*r*	*r*
1.	Exploitation t0	3.22	0.63	1									
2.	Exploration t0	2.54	0.66	− 0.454	0.000	1							
3.	Exploitation t1	2.64	0.71	0.432	0.000	− 0.249	0.019	1					
4.	Exploration t1	2.94	0.69	− 0.414	0.000	0.387	0.001	− 0.703	0.000	1			
5.	Process innovativeness	0.83	0.38	0.198	0.032	− 0.135	0.241	0.002	0.980	− 0.122	0.198	1	
6.	Process innovation radicalness	5.30	2.33	− 0.053	0.653	0.189	0.123	− 0.167	0.091	0.345	0.001	–	1

With regard to process innovations during the COVID-19-related school closures, 82.7% of the surveyed principals reported that such innovations had been implemented at their school during the school closure. In total, the surveyed principals reported 28 different types of innovations in teaching and instruction. This broad spectrum of innovations can be clustered into five areas: (a) digitalization, (b) supervision and support of students, (c) tasks and formats, (d) classroom-related student–teacher and student–student interaction, and (e) stakeholder (students, parents, teachers, principal) feedback. The five innovations most frequently mentioned with regard to teaching and instruction during the school closures were (a) the use of video conference systems (15.8%), (b) the introduction of digital learning platforms (13.3%), (c) the production of explanatory videos (11.2%), (d) the application of other kinds of digital learning approaches (9.1%), and (e) the introduction of weekly schedules for learning (7.1%).

Table 1 also reveals that the innovativeness of schools during school closure was significantly related to the exploitative activities of principals prior to closure (*r* = .198, *p* = .032) but not to their explorative activities, whether before or during the pandemic (*r* = − .135, *p* = .241 and *r* = − .122, *p* = .198), or to exploitative activities during school closure (*r* = .002, *p* = .830). The radicalness of innovations in teaching and instruction (*m* = 5.30) is significantly related to the principals’ explorative (*r* = .345, *p* = .001) and exploitative (*r* = − .167, *p* = .091) activities during school closure but apparently not to exploitative (*r* = − .053, *p* = .653) or explorative activities (*r* = .189, *p* = .123) prior to closure.

### Evaluation of measurement invariance

Measurement invariance is necessary to ensure that the measurement properties of our latent variables, exploration and exploitation, are stable over time and that changes are not a consequence of a change in the meaning and/or measurement of the measures. Hence, a series of successively more constrained models was conducted to evaluate the extent to which model assumptions are met. Table 2 shows the goodness-of-fit indexes of these models assessing longitudinal invariance.

**Table 2:** Fit indexes for invariance across time of exploration and exploitation measures

	CFI	ΔCFI	RMSEA	ΔRMSEA	SRMR	ΔSRMR	*χ*^2^(*df*)	Δχ^2^(*df*)
Configural invariance	1.000	–	0.000	–	0.017	–	5.36 (9)	–
Scalar invariance	1.000	0	0.000	0.000	0.019	0.002	7.37 (9)	2.01 (0)
Metric invariance	1.000	0	0.000	0.000	0.035	0.016	10.55 (11)	3.18 (2)
Strict invariance	1.000	0	0.000	0.000	0.040	0.005	13.72 (15)	3.17 (4)

Model 1 tests configural invariance, or whether the items show the same pattern of loadings on our constructs across measurement points and demonstrated a good fit (*χ*^2^ = 5.36; *df* = 9; CFI = 1.000; SRMR = .017; RMSEA = .000). Model 2 (*χ*^2^ = 7.37; *df* = 9; CFI = 1.000; SRMR = .019; RMSEA = .000) tested for metric invariance and thus whether the meaning of our latent variables were the same over time. No relevant differences in CFI (ΔCFI = 0), RMSEA (ΔRMSEA = 0), or SRMR (ΔSRMR = .002) were found. Further, ΔCFI did not exceed the .002 threshold, providing evidence of metric invariance. Model 3 tested for scalar invariance and thus assumed that intercepts are equivalent across time points. This model also fitted the data well (*χ*^2^ = 10.55; *df* = 11; CFI = 1.000; SRMR = .035; RMSEA = .000). Both ΔRMSEA and the ΔCFI were 0, with the latter thus below the .002 threshold, while ΔSRMR reached .016 and was thus well below the .030 threshold, suggesting that scalar invariance was supported. Finally, in Model 4, we tested for strict invariance and thus whether the residual variance was equivalent across time points. The model also fitted the data well (*χ*^2^ = 13.72; *df* = 15; CFI = 1.000; SRMR = .040; RMSEA = .000), and we found no relevant differences with regard to CFI (ΔCFI = 0), RMSEA (ΔRMSEA = 0), or SRMR (ΔSRMR = .005). Here as well, ΔCFI did not exceed the .002 threshold. Overall, these results provide substantial evidence for a very good level of invariance regarding our latent variables and allow for comparisons across measurement occasions.

### Cross-lagged panel study

**Fig. 1:**
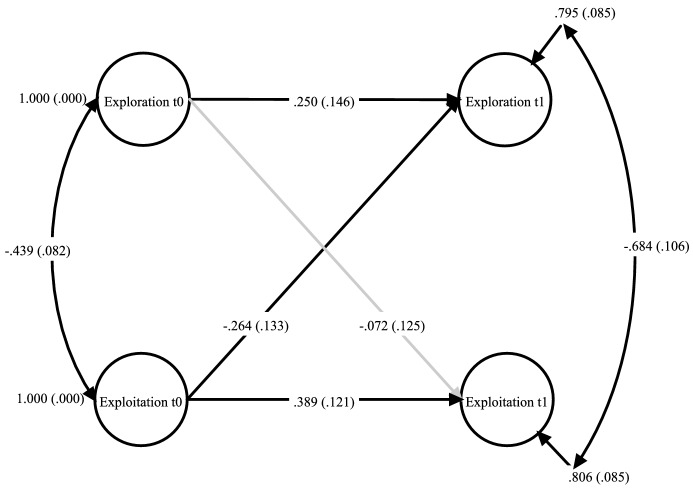
Latent cross-lagged panel model for principal exploration and exploitation with two time points. t0 = time point 1; t1 = time point two; standardized regression coefficients, standard errors in parentheses; non-significant paths grayed out

Next, we scrutinized the basic longitudinal CLPM (Model 1, see Fig. 1). For this purpose, we established autoregressive and predictive cross-lagged associations of principal exploitation and exploration over time. Further, we introduced latent correlations between exploitation and exploration for both measurement points in our model. This model fitted the data well (*χ*^2^ = 11.83; *df* = 14; CFI = 1.000; SRMR = .026; RMSEA = .000), and we found significant standardized path coefficients for all paths (*β*_exploration t0 -> exploration t1_ = .250, *p* = .087, *β*_exploitation t0 -> exploitation t1_ = .389, *p* = .001, *β*_exploitation t0 -> exploration t1_ = − .264, *p* = .048), with the exception of the path between explorative activities prior to COVID-19-related school closures and the exploitative activities during that time (*β*_exploration t0 -> exploitation t1_ = − .072, *p* = .564). The results demonstrate that principals’ ambidextrous activities depend upon their previous exploitative and explorative activities and that principals reproduce their familiar learning patterns even in times of crisis. The results further point to an exploitation bias, as principal exploitation before COVID-19 increased the likelihood that exploitation was performed again during the school closures and hindered potential increases in explorative activities during that time. Thus, hypotheses H2a, H2b, and H3b are confirmed and H3a is rejected.

In a next step, we introduced process innovativeness (Model 2a) and process innovation radicalness (Model 2b) during the school closure in the previous model to test whether principal ambidexterity was associated with process innovations in schools during the COVID-19 pandemic. Thus, we scrutinized autoregressive and cross-lagged associations and regressions between exploitation and exploration at both times and a school’s innovativeness respectively innovation radicalness in teaching and learning during the school closure in the previous model. Regarding innovativeness (Model 2a, *χ*^2^ = 14.66; *df* = 18; CFI = 1.000; SRMR = .026; RMSEA = .000), we only found a statistically significant association of principals’ exploitative activities prior to the COVID-19 pandemic with the innovativeness of schools (*β*_exploitation t0 -> innovativeness t1_ = .243, *p* = .025). Hence, schools where principals focused mainly on efficiency and refinement before the pandemic began were more likely to innovate teaching and instruction during the pandemic. All other paths (*β*_exploration t1 -> innovativeness t1_ = .050, *p* = .780, *β*_exploitation t1 -> innovativeness t1_ = −.147, *p* = .360, *β*_exploration t0 -> innovativeness t1_ = .061, *p* = .594) were not related to the innovativeness of schools. As we observed no associations between exploration and exploitation during the COVID-19 pandemic and the innovativeness of schools during that time, we did not test for indirect (longitudinal) effects.

Regarding the innovation radicalness in teaching and instruction (Model 2b, *χ*^2^ = 26.93; *df* = 18; CFI = .971; SRMR = .033; RMSEA = .032), we again discovered one statistically significant association. With regard to the degree of novelty of the innovations created during school closure, we found a significant association between explorative activities during the second measurement point and innovation radicalness in teaching and instruction (*β*_exploration t1 -> innovation radicalness t1_ = .393, *p* = .053). Here, too, all other direct paths (*β*_exploitation t0 -> innovation radicalness t1_ = .170, *p* = .256, *β*_exploration t0 -> innovation radicalness t1_ = .084, *p* = .526, *β*_exploitation t1 -> innovation radicalness t1_ = .081, *p* = .683) were not significantly related to the innovativeness of schools during the pandemic. As exploration activities during the pandemic were associated with innovation radicalness during that time and we observed significant relations with both explorative and explorative activities of principals before the pandemic started, we evaluated possible indirect effects in this regard, following Preacher and Kelley (2011). Although the coefficients were comparatively large, no statistically significant relationship could be demonstrated (*β*_exploitation t0 -> exploration t1 -> innovation radicalness t1_ = − .102, *p* = .200, *β*_exploration t0 -> exploration t1 –> innovation radicalness t1_ = .096, *p* = .229). Consequently, H4 was rejected and H5 confirmed.

**Fig. 2:**
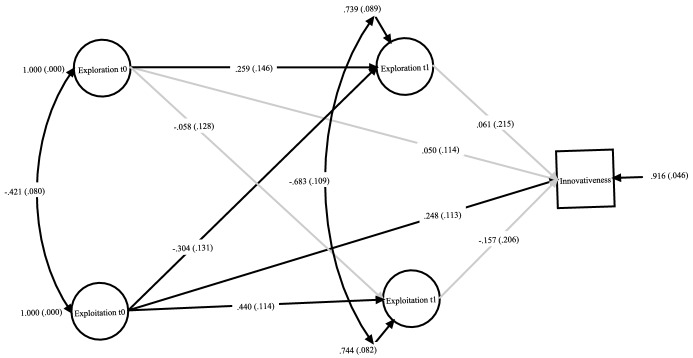
Latent associations of principals’ exploration and exploitation activities with schools’ innovativeness in teaching and instruction. *Note* t0 = time point 1; t1 = time point two; standardized regression coefficients, standard errors in parentheses; non-significant paths grayed out. All variables controlled for school size, school type and rural–urban split

In a final step, we controlled all model variables—exploitation at t0 and t1, exploration at t0 and t1, and innovativeness (Model 3a) and innovation radicalness (Model 3b)—by school size, school type, and urban–rural character to rule out other possible causes for the observed relationships (Hamaker et al., 2015; Little, 2013). Model 3a (see Fig. 2) also fitted the data well (*χ*^2^ = 33.70; *df* = 38; CFI = 1.000; SRMR = .025; RMSEA = .000). When controlled for those contextual variables, all standardized path coefficients show tendencies similar to the earlier model (*β*_exploitation t0 -> innovativeness t1_ = .248, *p* = .028, *β*_exploitation t1 -> innovativeness t1_ = − .157, *p* = .445, *β*_exploration t0 -> innovativeness t1_ = .050, *p* = .661, *β*_exploration t1 -> innovativeness t1_ = .061, *p* = .776). Again, we did not calculate any indirect effects. Regarding our control variables, we found that school size (*r*_school size -> exploration t0_ = − .232, *p* < .001) and rural–urban split were significantly negatively associated with principals’ explorative activities (*r*_rural urban split -> exploration t0_ = − .114, *p* = .048) and significantly positively associated with their exploitative activities (*r*_school size -> exploration t0_ = .121, *p* = .037, *r*_rural urban split-> exploration t0_ = .141, *p* = .013) prior to school closure. We did not discover statistically significant associations between school type and any model variable or associations between the control variables and exploitation, exploration, or innovativeness during the pandemic (*p* > .10).

**Fig. 3:**
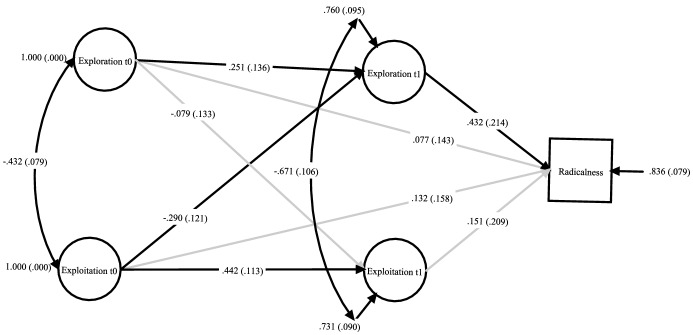
Latent associations of principals’ exploration and exploitation activities with schools’ innovation radicalness in teaching and instruction. *Note* t0 = time point 1; t1 = time point two; standardized regression coefficients, standard errors in parentheses; non-significant paths grayed out. All variables controlled for school size, school type and rural–urban split

Model 3b also fitted the data well (*χ*^2^ = 47.15; *df* = 38; CFI = .976; SRMR = .028; RMSEA = .022; see Fig. 3) and demonstrated the overall stability of the model parameters. Even when we controlled for potential contextual confounders, principal exploration during the COVID-19 pandemic had a statistically significant effect on innovation radicalness in teaching and instruction during school closure (*β*_exploration t1 -> innovation radicalness t1_ = .432, *p* = .044), whereas we still could not detect any significant effects on this for exploitation (*β*_exploitation t0 -> innovation radicalness t1_ = .132, *p* = .469) and exploration (*β*_exploration t0 -> innovation radicalness t1_ = .077, *p* = .591) prior to the pandemic or for exploitative activities during the pandemic (*β*_exploitation t1 -> innovation radicalness t1_ = .151, *p* = .392). All covariates included in the model were not statistically significantly related to innovation radicalness (*p* > .100). We also found no indirectly mediated effects in this model that would have withstood a significance test (*β*_exploitation t0 -> exploration t1 -> innovation radicalness t1_ = − .133, *p* = .166, *β*_exploration t0 -> exploration t1 -> innovation radicalness t1_ = .110, *p* = .181). Thus, even with the addition of time-invariant control variables, H4 was rejected and H5 was confirmed.

## Limitations

Despite the strengths of our study, which featured a representative panel of principals based on a random sample, a few limitations deserve mention: First, we are aware that it was impossible in our study to control properly for unit effects and estimate robust autoregressive terms, as we only applied two measurement occasions, although we integrated time-invariant control variables in our analyses (Zyphu et al., 2020a, 2020b). Second, our analyses rely on principal self-reports and thus misreporting cannot be completely ruled out. In this context, responses of school leaders to the survey given during the spring of 2020 could reflect a general societal state that was marked by uncertainty and volatility. In terms of measuring innovation, we think we have gone a good distance in applying the OECD’s Oslo guidelines for collecting, reporting, and using data on innovation in an educational setting. However, even if such surveys are considered state of the art by some (OECD, 2014), non-reactive measures would be helpful in determining which innovations actually emerge in the field. Hence, future research would do well to explore innovations through measurements decoupled from surveys. Third, although the concepts of exploitation, exploration, and ambidexterity are fairly a well-researched topic outside the educational sector, there is a lack of studies that would allow us to draw conclusions about the extent to which the concept is generalizable and, more importantly, transferable to the school setting. Since we believe this is a concept that can help provide new insights into how school leaders affect educational change, especially in light of the increasing dynamics and uncertainties in education, further studies that follow our lead would be desirable.

## Discussion and conclusion

The central purpose of this study was to investigate whether and how school leaders in Germany adapt their exploitation and exploration activities to a turbulent environment and whether and how these two complementary, mutually affecting knowledge strategies were associated with school-wide innovation efforts in teaching and instruction in German schools during the COVID-19 pandemic.

The findings broadly show that the concepts of exploitation, exploration, and ambidexterity can be applied to the school setting. Thus, our study confirms assumptions and findings that have already been demonstrated in other fields of research: Leaders dynamically adapt their knowledge strategies to the context (Germain, 1996; Koberg et al., 2003), they increasingly use explorative strategies in times of crisis (Lavie et al., 2010), and those activities have an impact on the innovativeness of their institutions (de Visser & Faems, 2015). In this respect, we were able for the first time to demonstrate path dependency, exploration bias and a relationship between a school leader's exploration and exploitation and school-wide innovation and innovation radicalness in teaching and instruction.

Further, we were able to show that the school leaders in our sample used a different approach in turbulent, uncertain environments than in secure and certain environments. We found that these principals’ strong focus on “refinement, choice, production, efficiency, selection, implementation and execution” (March, 1991, p. 71) in more secure environments (pre-COVID-19) seemed to inhibit creativity, flexibility, risk-taking, and experimentation in uncertain times (COVID-19). For more profound innovations to emerge that have the potential to bring radical change, our analysis suggests that the “pursuit of new knowledge” (Levinthal & March, 1993, p. 105) is needed.

Accordingly, our research suggests that those schools that innovatively addressed the COVID-19 pandemic were schools whose leaders were able to quickly shift between the two modes of exploitation and exploration, or, as Tushman and O’Reilly (1996, p. 11) put it, who proved to be jugglers of knowledge when schools were closed. In this respect, our study makes it clear that during the pandemic, it was necessary “to navigate a different course, to create new pathways through the disruption” (Harris & Jones, 2020, p. 246) in other words to have the capacity to quickly shift modes.

In terms of the (changing) role of school leaders over the course of COVID-19, our findings are consistent with those of other studies and show that flexibility, creativity and changing priorities were central to school leaders in the early stages of the pandemic (Beauchamp et al., 2021; Huber & Helm, 2020; Longmuir, 2021; McLeod & Dulsky, 2021; Thornton, 2021). However, unlike many other studies, we had the advantage of longitudinal data, with the first measurement point prior to the pandemic outbreak, so we were able to assess changes over time and consequently evaluate the concept of ambidexterity as a dynamic managerial capability (Papachroni & Heracleous, 2020) of school leaders.

In this way, we were able to explore how school leaders dealt with the fundamental tension between efficiency and flexibility, and clarify the microfoundations of innovative performance of schools in dynamic environments (Eisenhardt et al., 2010). Results are unambiguous: while previous studies suggested that the “capabilities of school principals to foster conditions that support effective teaching and learning practices … are at the core of effective school leadership” (Lai, 2015, p. 70), our research makes clear that a certain degree of flexibility is also required to apply those capabilities in dynamic, changing contexts.

In summary, our investigation demonstrates in many ways the practical relevance of the exploitation-exploration distinction in relation to school leadership and innovation in teaching and instruction during the COVID-19 pandemic in the German context. It makes clear, that schools and their leaders must continuously maintain and improve upon the status quo while always being prepared for the unexpected. Hence, for a school to thrive in challenging circumstances, thinking outside the box and being able to dynamically switch modes and adapt appear to be crucial skills in a principal.

Although it is currently rather unclear how to promote individual ambidexterity (Turner et al., 2015), the key prerequisite for successfully dealing with the demands of exploration and exploitation seems to be a paradoxical mindset (Smith & Tushman, 2005) that enables school leaders to use these two knowledge strategies not as disjunct either/or trade-offs but rather as interwoven both/and approach (“How can you do A without letting B be?”, Smith et al., 2016). Therefore, in our opinion, in practice a special demand must be placed on the formal qualifications of school leaders, who must constantly be aware of the relevance of exploration despite the strong pull toward exploitation that is often exerted by their day-to-day tasks and administrative demands.

In terms of research, first and foremost it should be pointed out that, in the entire field of research on ambidexterity, our study is only one of two to investigate the presumed relationship between individual ambidexterity and an organization's innovation outcome (Pertusa-Ortega et al., 2021) and the only one so far to empirically examine this in the context of schools. Thus, in summary, it thus appears worthwhile to explore alternative and novel ways of researching educational leadership and school improvement and innovation in turbulent times. Since ambidexterity research is scarce in the field of education, there are a number of questions that need to be answered in the future. As our study suggests that a schools’ innovativeness and innovation radicalness is significantly affected by a school leaders’ capability to handle the paradox of exploitation and exploration, the most pressing question is probably the one about the modes and mechanisms of action. Theoretically as well as empirically, the following question has to be addressed: How and in what ways does the ambidextrous behavior of school leaders contribute to changing schools?

Here, it seems particularly important to focus on the role of teachers, as both research on leadership in schools (Leithwood et al., 2020) and research on ambidexterity (Mom et al., 2019) have shown that it is the individuals in a school who contribute to its success through unique top-down and bottom-up pathways, and furthermore, that the central position of the principal in a school's social network is an important factor in fostering innovation in schools (Moolenaar et al., 2010). Given that our study covers only a short, albeit disruptive and highly dynamic, period in which, moreover, the social contacts of school staff, e.g., as a result of home offices, were often of a different nature than in the pre-COVID-19 period, it would be worthwhile to explore the extent to which our findings can be replicated in non-turbulent periods, taking into account the dynamics of principal ambidexterity and educational innovation as presented here as well as the multilevel nature of school leadership (Boyce & Bowers, 2018; Da’as, 2021; Pietsch et al., 2019).

## Appendix 1

### Items used to measure principal exploitation and exploration

For measuring principal exploitation and exploration, we use and adept items developed by Mom et al. (2009).

t0: To what extent did you, during the last 12 months, engage in work-related activities that can be characterized as follows:

t1: To what extent did you, since your school closed due to COVID-19, engage in work-related activities that can be characterized as follows:

#### Exploitation

Activities which you can properly conduct by using your present knowledge.*

Activities of which it is clear to you how to conduct them.*

Activities that serve to ensure the smooth running of everyday business.^1^

Activities which you carry out as if it were routine.^2^

#### Exploration

Activities requiring you to learn new skills or knowledge.*

Activities of which the associated yields or costs are currently unclear.*

Activities focusing on strong renewal of products/services or processes.^1^

Activities requiring quite some adaptability of you.^2^

- All items were measured on a four-point scale (1 = to a small extent to 4 = to a large extent).

*Anchor Items, ^1^only used at t0, ^2^ only used at t1.

## Appendix 2

### Items used to measure innovativeness and innovation radicalness

For measuring innovativeness and innovation radicalness, we adapted and expanded the survey design of the European Community Innovation Survey (CIS, Behrens et al., 2017).

#### Introduction

Many schools have had to make changes and innovations in everyday school life as a result of school closures. Corresponding innovations can affect the school's processes, organization or social structure, among other things:

- *Process innovations* comprise new or noticeably changed processes with regard to the pedagogical work of the school (e.g., instruction and/or teaching);
- *Organizational innovations* include the structural development or redesign of the school's internal work processes or work organization (e.g., with regard to conferences and staff meetings);
- *Social innovations* include the creation of new or improved conditions or measures for school employees (e.g., with a view to cooperation within the teaching staff);
- *Service innovations* include the provision of new and/or optimized services (e.g., changed contact options for students and parents with regard to questions).

#### Measurement of innovativeness

Were any innovations introduced at your school during the school closure in the following areas?

- Process innovations
- Organizational innovations
- Social innovations
- Service innovations

- All items were measured on a binary scale (0 = no; 1 = yes).

#### Measurement of concrete innovations (respectively, for 2a, 2b, 2c and 2d)

What were the main innovations in this area during the school closure? Please give a maximum of three examples.

- Open item format.

#### Measurement of innovation radicalness (respectively, for 2a, 2b, 2c and 2d)

Are these changes incremental (improving and/or supplementing and/or adapting what already exists) or radical (introducing something completely new) for your school?

- Item measured on a ten-point scale (1 = incremental to 10 = radical).

## Appendix 3

### Exemplary Mplus input

Title: Latent Cross-Lagged Panel Model with Regression(s) on Innovation Radicalness

Data: File is ambdext0t1.dat; ! file name

Format is f4.0, 11f2.0, f4.0; ! format statement

Variable: Names are ! Variable names

id ! principal ID

f10_1 f10_2 ! indicators for exploitation t0

f10_4 f10_5 ! indicators for exploration t0

t1f9_1 t1f9_2 ! indicators for exploitation t1

t1f9_4 t1f9_5 ! indicators for exploration t1

t1f8b_1 ! innovation radicalness

f2 f3 nf4; !control variables

Use variables are ! Names of analysis variables

f10_1 f10_2 f10_4 f10_5t1

t1f9_1 t1f9_2 f9_4 t1f9_5

t1f8b_1 f3 nf4 ws foe as;

ID Variable is id; ! Name of principal ID variable

Missing are f10_1-t1f9_5 (99) ! Missing codes

f2 (99) f3 (99) nf4 (9999) ! Missing codes

t1f8b_1 (99); ! Missing codes

!Recoding the school type variables into dummy variables

DEFINE:

gs = 0; ws = 0; foe = 0; as = 0; ! gs = primary, ws = secondary, foe = special needs, as = other school

if (f2 eq 1) then gs = 1; if (f2 eq 2) then ws = 1; if (f2 eq 3) then ws = 1; if (f2 eq 4) then ws = 1;

if (f2 eq 5) then ws = 1; if (f2 eq 6) then ws = 1; if (f2 eq 7) then foe = 1;if (f2 eq 8) then bs = 1;

if (f2 eq 9) then as = 1;

!Imputation model

Data Imputation:

Impute =

f10_1 f10_2 f10_4 f10_5 t1f9_1 t1f9_2 t1f9_4 t1f9_5 t1f8b_1 f3 nf4 ws(c) foe(c) as(c);

Ndatasets = 100;

Save = ambdex1*.dat;

!Latend cross-lagged model with regression on innovation radicalness

Model:

exploit0 by f10_1 f10_2; ! Factor exploitation t0

explort0 by f10_4 f10_5; ! Factor exploration t0

exploit1 by t1f9_1 t1f9_2; ! Factor exploitation t1

explort1 by t1f9_4 t1f9_5; ! Factor exploration t1

explort0 with exploit0; ! Correlation of exploitation and exploration t0

explort1 with exploit1; ! Correlation of exploitation and exploration (residuals) at t1

exploit0 with f3 nf4 ws foe as; ! Correlation of exploitation and control variables at t0

explort0 with f3 nf4 ws foe as; ! Correlation of exploration and control variables at t0

explort1 on explort0 (A); ! Autoregression between exploration t0 and t1

explort1 on exploit0 (B); ! Cross-lagged regression between exploitation t0 and exploration t1

explort1 on ws foe as f3 nf4; !Regression of control variables on exploration t1

exploit1 on exploit0; ! Autoregression between exploitation t0 and t1

exploit1 on explort0; ! Cross-lagged regression between exploration t0 and exploitation t1

exploit1 on ws foe as f3 nf4; !Regression of control variables on exploitation t1

t1f8b_1 on exploit0 explort0 exploit1 ! Regression of exploitation t0, t1 and exploration t1 on

!innovation radicalness t1

t1f8b_1 on ws foe as f3 nf4; ! Regression of control variables on innovation radicalness t1

t1f8b_1 on explort1(C); ! Regression of exploration t1 on innovation radicalness t1

!Estimation of standardized indirect effects

!Formula: path1*path2*SDx/SDy, see Preacher and Kelley (2011)

Model constraint:

new (plor ploi);

Plor = A*C*0.358/2.330; ! Path exploration t0 on innovation radicalness via exploration t1

Ploi = B*C*0.505/2.330; ! Path exploitation t0 on innovation radicalness via exploration t1

Output: Tech1 Tech4 Tech8 TECH9 Standardized; ! Output command
